# Engaging with research ethics in central Francophone Africa: reflections on a workshop about ancillary care

**DOI:** 10.1186/1747-5341-7-10

**Published:** 2012-08-06

**Authors:** Tomi Tshikala, Bavon Mupenda, Pierre Dimany, Aime Malonga, Vicky Ilunga, Stuart Rennie

**Affiliations:** 1 Kinshasa School of Public Health, Kinshasa, Democratic Republic of Congo, Kinshasa, Congo; 2 Center Interdisciplinaire de Bioéthique pour l’Afrique Francophone (CIBAF), Kinshasa School of Public Health, Kinshasa, Democratic Republic of Congo, Kinshasa, Congo; 3 UNC Center for Bioethics, Universtity of North Carolina at Chapel Hill, Chapel Hill, NC, USA; 4 Department of Social Medicine, University of North Carolina at Chapel Hill, Chapel Hill, NC, USA

**Keywords:** Research ethics, Developing countries, Francophone Africa, Ancillary care, Medical education

## Abstract

Research ethics is predominantly taught and practiced in Anglophone countries, particularly those in North America and Western Europe. Initiatives to build research ethics capacity in developing countries must attempt to avoid imposing foreign frameworks and engage with ethical issues in research that are locally relevant. This article describes the process and outcomes of a capacity-building workshop that took place in Kinshasa, Democratic Republic of Congo in the summer of 2011. Although the workshop focused on a specific ethical theme – the responsibilities of researchers to provide health-related care to their research participants – we argue that the structure of the workshop offers a useful method for engaging with research ethics in general, and the theme of ancillary care encourages a broad perspective on research ethics that is highly pertinent in low-income countries. The workshop follows an interactive, locally driven model that could be fruitfully replicated in similar settings.

## Introduction

Despite the globalization of health research involving human participants, ethical reflection on research has remained to some extent parochial. It is generally accepted that bioethics as a field of inquiry emerged in the United States, and although there are some important exceptions, the field continues to be strongly marked by its origins: the most prestigious and influential journals, bioethics centers, graduate programs, and influential funding institutions are Anglophone and located in the United States, Canada, Australia and Western Europe. [1] Over the last decade, there has been a perceived need for bioethics to be practiced in low-resource (and not necessarily Anglophone) settings by local stakeholders, and a number of important recent initiatives have global bioethics capacity-building as their mission. Such initiatives are faced with the thorny problem of how to cultivate interest and skills in bioethics in low-resource settings while avoiding the imposition of foreign conceptual frameworks and ensuring that the ethical issues are locally relevant. In what follows, we describe a way of responding to these challenges in the form of a workshop that was held in the Democratic Republic of Congo in May 2011. We argue that the structure of the workshop is a highly interactive way of engaging with research ethics issues, and the issue of ancillary care is a useful point of entry into a wide array of ethical concerns relevant to research in resource poor settings.

## Background

The Democratic Republic of Congo (DR Congo) is the third largest country in Africa with a population of approximately 77 million persons. Despite its rich natural resources, this former Belgian colony has a long history of exploitation and political violence that continues to this day. The vast majority of citizens in the DR Congo live near or below the poverty line, life expectancy (55.3 years) is one of the shortest in the world, and the rate of infant mortality (78.4 deaths/1000 live births) is one of the world’s highest. [2] Health-related research in the country focuses primarily on infectious diseases, especially HIV/AIDS, tuberculosis, monkey pox, malaria, as well as social science research on issues such as street children in Kinshasa and sexual violence in the Eastern Congo. The University of North Carolina at Chapel Hill has conducted public health research in the DR Congo since 2001, where the flagship projects have been operational research involving delivery of HIV/AIDS related care, treatment and prevention services to pregnant women, their partners and other household members.

In 2004, with the support of a NIH/Fogarty bioethics capacity building grant, the University of North Carolina at Chapel Hill (UNC) and the Kinshasa School of Public Health (KSPH) established the Centre Interdisciplinaire de Bioethique pour L’Afrique Francophone (CIBAF). In order to enhance sustainability, this project aims to build capacity in bioethics at individual and institutional levels by training a core of Congolese professionals and integrating CIBAF into the educational program and administrative structure of the KSPH. CIBAF regularly conducts bioethics capacity-building activities within the KSPH, at neighboring University of Kinshasa, national health agencies within Kinshasa (such as the National AIDS Control Board) as well as regional outreach to Lumumbashi and Kisangani, and international outreach to Burundi and Madagascar. CIBAF members and KSPH faculty recently took a leading role in the development of the first research ethics guidance for the DR Congo, to be published by the Ministry of Health in 2012.

In 2007, the Congolese members of the UNC/DRC research team created their own working group to discuss ethical issues encountered in research, the *Groupe Interproject de Reflexion et d’Intervention en Ethique* (GIRIE). The most prevalent ethical issue identified by the group was ‘ancillary care responsibilities’, i.e. whether and how to respond when research participants have significant health problems that are not directly related to the research. What, if anything, should researchers (or others) do in such cases? This is a common situation in settings with high disease burdens and weak health infrastructures, but has only gained some measure of scholarly attention in the last few years. [3-8] For this reason, members of GIRIE, CIBAF and KSPH faculty decided to hold a 3-day workshop on ancillary care in health-related research in the DR Congo. To our knowledge, this was the first workshop in Francophone Africa to be devoted to this theme.

### Workshop composition, objectives and format

#### Workshop composition

A limited number of participants were invited from key health-related research stakeholders in the DR Congo, including: members of CIBAF; faculty from the Catholic University of the Congo; members of clinical ethics committees; faculty from the Kinshasa School of Public Health and the University of Kinshasa; representatives of local health NGOs; members of UNC/DRC research teams; representatives of local pediatric clinics; representatives of the National AIDS Control Program. A modest number of invitees (n = 30) were deliberately chosen in order to keep discussions focused, facilitate interactive sessions and enable all participants to express their viewpoints.

### Workshop objectives

Members of GIRIE and CIBAF constituted the workshop organizational committee and established the following six objectives:

· To develop a working definition of bioethics and ancillary care in general, in relation to the specific context of health-related research in the Congo

· To critically assess different ways of analyzing the ethical problem of ‘ancillary care’, particularly through case studies of ancillary care issues in resource-poor countries

· To elaborate a model and materials to teach the issue of ancillary care

· To present a report to the DR Congo’s national health ethics committee

· To develop a communal ‘workshop message’

· To craft a manuscript on the process and outcomes of the workshop and disseminate results to interested parties within Francophone Africa

### Workshop format

To realize the workshop objectives, the organizational committee decided on a combination of didactic and interactive components. The didactive components consisted of formal presentations followed by discussion with all workshop participants. The interactive sessions involved specific ‘hands-on’ activities among small work groups related to the ethics of ancillary care.

### Didactic components

The committee felt that although some of the audience had some knowledge about research ethics through earlier CIBAF initiatives, discussion of basic concepts and distinctions were necessary to develop a shared language and to minimize potential misunderstandings. An introductory presentation offered a working definition of ethics (and bioethics), and explored the relationship between ethics, common morality, existing law and religious faith. Experiences of researchers with ancillary care in the DR Congo were presented, including a number of case studies for later discussion. The model of ethical decision-making by Belsky and Richardson on the ancillary care responsibilities of researchers was presented in some detail (see below). We primarily utilized Belsky and Richardson’s article ‘Medical researchers’ ancillary care responsibilities’ (2004) in the *British Medical Journal*, because we felt the article was clear, concise and accessible to a broad audience of health professionals, with or without a background in philosophy. Other presentations focused on the current state of international policies and guidelines in regard to ancillary care; the possible role of ethics committees in regard to ancillary care; ethical issues related to ancillary care, in particular incidental findings and post-research obligations. Incidental findings are unsuspected health conditions of research participants, often discovered through diagnostic tests; the discovery of these conditions raise questions about researcher’s responsibility for care. When research ends, participants may still need the health care that was provided during the study, and here again the existence and the extent of the researcher’s responsibilities come into question. While incidental findings and post-research benefits do not properly fit the definition of ancillary care, they raise similar issues and can provide helpful counterpoints.

### Interactive components

The workshop participants were divided into three working groups, and were tasked with activities related to the issues mentioned above. Participants were requested to critically reflect on what ‘ethics’, ‘morality’, and ‘ancillary care’ means in the context of the DR Congo, as well as what could be reasonably expected of researchers (or research funders) in meeting ancillary health needs of research participants. There was much heated debate about whether certain case studies were indeed instances of ancillary care, and if so, what the responsibilities of researchers might be in such cases. Crucially, all three working groups were asked to design an ethical decision-making model of ancillary care in response to the Belsky and Richardson model, and then all participants attempted to merge all three into one single model. Participants were then tasked with the development of a common message about ancillary care, incorporating the diverse ethical considerations discussed during the workshop.

### Workshop outcomes

#### Criticisms of the Belsky and Richardson model

In building an analytic model on ancillary care, Belsky and Richardson make a crucial distinction between questions of scope and strength:

· Scope: is this health condition of the research participant something the researcher is responsible for?

· Strength: how strong is the responsibility of the researcher for the health condition of the participant? [9]

To guide answers to the question of scope, Belsky and Richardson propose that we look at the aspects of his/her health the participant has placed under the researcher’s care (‘entrusted’) when agreeing to join the research study. How this question is answered depends on the nature of the research question, the specific procedures involved, and what the participant agreed to in the consent process. For example, in a malaria prophylaxis study, a participant’s dental or psychological needs would presumably lie outside the scope of what the malaria researchers are responsible for.

To guide answers to the question of strength, Belsky and Richardson propose other criteria: vulnerability, dependence, depth, gratitude and cost. By *vulnerability*, the question is how badly the research participant would be affected if he/she did not receive the needed care; the worse off the participant would be, the stronger the responsibility. *Dependence* refers to access to care: the less able the participant is to access needed care outside the research setting, the stronger the responsibility. *Depth* refers to the quality of the relationship between researchers and research participants. Some studies are short and involve few interactions, unlike longitudinal and participatory studies. According to Belsky and Richardson, the deeper the relationship, the stronger the responsibility to provide ancillary care, all other things being equal. *Gratitude* refers to the degree to which researchers are indebted to the participants to carry research on a particular research question, including indebtedness arising from the inconvenience or risks the participants may face or endure. *Cost* refers to the human and financial resources involved in provision of ancillary care. If the costs are too great relative to the study budget, providing ancillary care would compromise the conduct of the scientific research, and therefore the higher the cost, the weaker the strength of the responsibility. Using the parameters of scope and strength, progress in determining the responsibility for providing ancillary care in particular studies can be made.

Participants at the Kinshasa workshop expressed criticisms and concerns about both parameters. In regard to scope, participants could appreciate that scope criteria must exist in order for research projects not to be overwhelmed by ancillary care needs of participants. Participants also recognized that when research participants are in urgent medical need, have no alternative means of help, and providing care would involve no great sacrifice on the part of the researchers, there is a ‘duty of rescue’ (though participants tended to conceptualize this in terms of ‘humanity’ or ‘solidarity’) even if the urgent condition lies outside the scope of the research. On the other hand, participants were concerned about the outcome of applying the criterion strictly. Belsky and Richardson’s model seems to justify no provision of ancillary care when a serious but not urgent medical need (i.e. a need not clearly falling under a ‘duty to rescue’) lies outside the scope of the research. Participants in the Kinshasa workshop argued that providing literally nothing in these ‘grey zone’ cases in resource-poor settings would be unacceptable. In addition, providing nothing when local ancillary care needs are serious (but not urgent and life-threatening) and could be dealt with to some extent (e.g. by referrals or by provision of basic information) at low-cost seems irresponsible, even if those needs (according to Belsky and Richardson’s scheme) lie outside the scope of the researcher’s responsibility. This criticism resonates with similar concerns in the recent literature. [5] Participants also recognized that it is sometimes difficult to determine whether ancillary care needs in specific cases lie inside or outside the scope of researcher responsibility, and Belsky and Richardson’s model does not always provide guidance for such cases. In a study on HIV transmission in Kinshasa [10], an HIV negative woman reported being physically abused by her HIV-positive husband. The husband refused to use condoms and hit his spouse whenever she refused to have unprotected sexual relations. He also threatened to hit the child of his spouse (from a previous marriage) if she refused to have sex with him. She was afraid to leave her husband because he bought food for the household and paid for their children’s school fees. Given the known familial problems among HIV sero-discordant couples, are researchers responsible for providing psychosocial support?

Workshop participants also raised questions about the strength criteria in the Belsky and Richardson model. There was some concern about basing the strength of ancillary care responsibilities on the ‘depth’ of the relationship between researchers and research participants, because participants in short-term studies may have significant health needs. Thinking that the responsibility is less in such cases was thought akin to thinking that distance or country membership is a legitimate ethical reason to care less about the suffering of others. On the question of cost, participants were divided: at the extreme, if the cost of providing ancillary care would be overwhelming, that would be a significant reason not to provide them. But there was much debate on how much cost considerations should be factored into such decisions, because cost is commonly cited (by foreign research agencies) as the most valid reason not to provide ancillary care. In addition, participants pointed out that provision (and even the offer) of ancillary care can be inappropriate for cultural and/or religious reasons: local religious groups may not accept certain health interventions, even if providing them is justified on Belsky and Richardson’s model [11].

### Alternative model for ancillary care: a work in progress

The perceived need for an alternative model for ancillary care arose from conflicts between Belsky and Richardson’s model and the moral intuitions of those participating in the workshop. In certain cases, Belsky and Richardson’s model made determinations of moral responsibility that diverged from what workshop participants believed to be correct. For example, when the health condition of a research participant is unrelated to the research question or study procedures, Belsky and Richardson’s model suggests that researchers have no obligation to assist the participant, even if the condition is quite serious, could pose health risks to others, or would be simple and affordable to treat. There was an overall impression that Belsky and Richardson’s model did not fully incorporate the range of factors and variables that could go into such decision-making, particularly in resource poor settings. Some of these factors included: the impact of providing or not providing ancillary care on the local community or relatives of the research participant; the type of health condition involved, and whether it is chronic or acute; the negative impacts on research (including future research) of providing ancillary care; whether provision of ancillary care is a responsibility when there are reasons to believe that the care is not locally sustainable. Participants felt that the challenge was to accommodate these myriad considerations and complications when developing an alternative model of ancillary care responsibilities more in keeping with local views about equity and solidarity.

Participants were often of two minds: concerned about placing excessive burdens on health researchers in resource-poor countries, and worried about neglecting persons with significant health needs who have nowhere else to turn. Any model of ancillary care responsibilities must deal with this tension, but it was felt that cost was permitted to play an overly dominant role in Belsky and Richardson’s model at the expense of vulnerability. The idea was floated that not all strength factors are created equal: serious vulnerability and dependence of research participants creates a *prima facie* obligation to provide care, even when the needs may not relate to the research question. Participants argued that if cost is to be included in the strength criteria, then funders of research in developing countries must accept to some degree that provision of ancillary care is integral to the conduct of ethical health research in developing countries, particularly when past research has often had little sustainable impact on health in local communities. Local ethics committees need to be empowered to factor ancillary care issues into account when reviewing research proposals. Unless there is serious provision and oversight, cost factors in practice are likely to eclipse all other considerations.

Efforts to construct an alternative model of ancillary care responsibilities led to the articulation of a novel idea. The extent and complexity of health needs in low-resource countries encouraged participants to think in terms of ‘ancillary services’ integrated into local health systems rather than ancillary care needs provided by researchers to individual participants. When specific ancillary care needs can be clearly anticipated, researchers should work with local communities to create or bolster existing services to meet those needs, and the service costs should be reflected in the research budget. While researchers cannot be expected to replace local health services, workshop participants generally felt that it is reasonable to expect some contribution (5-10% of the study budget) to foreseeable ancillary care needs in low-resource settings.

**Figure 1:**
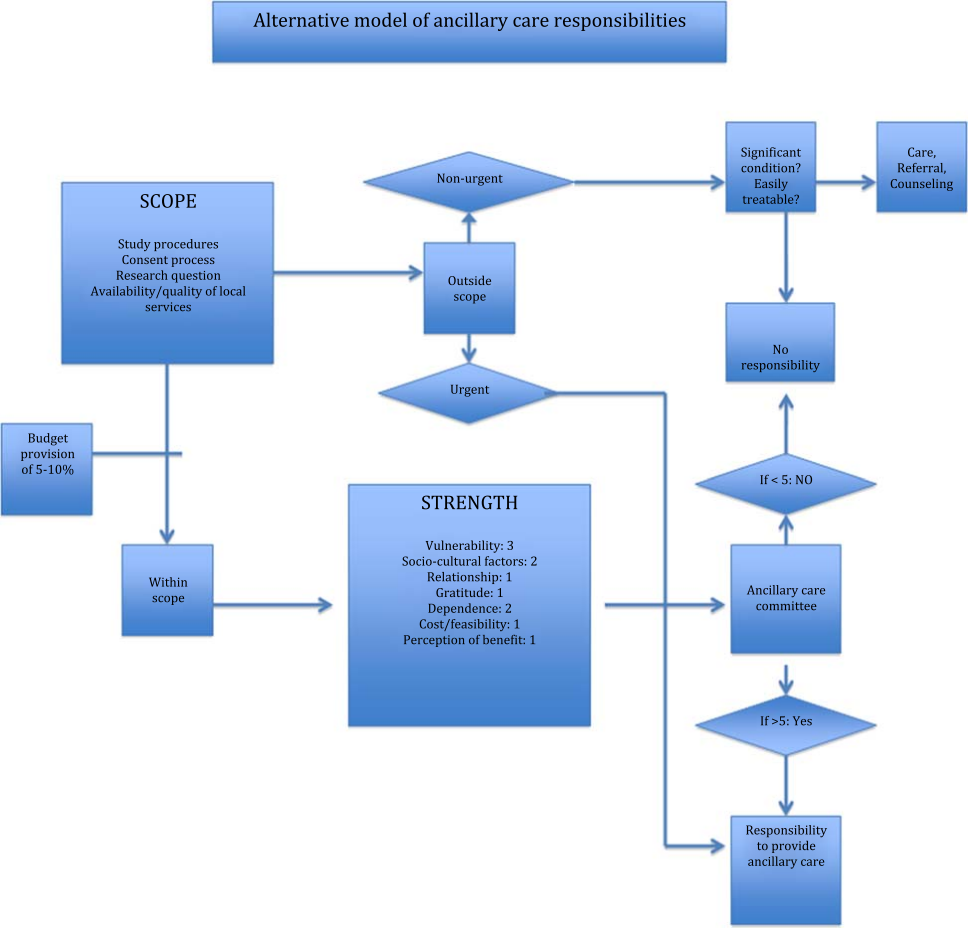
Alternative model of ancillary care responsibilities.

Determining the strength of research responsibilities requires identifying relevant considerations and some idea how to weigh different considerations against one another. Workshop participants found these challenges particularly vexing. Participants generally agreed with the considerations proposed by Belsky and Richardson, but added elements such as socio-cultural factors and the perception of benefit by the research participant (see Figure 1). Some participants proposed a 10-point scale to measure strength of researcher responsibility, where the vulnerability of the participant features as the weightiest consideration, relative to others (such as cost). Other participants argued that the ‘weighing’ was metaphorical and quantifying the considerations would lead only to a false impression of objectivity. On the other hand, participants were not entirely comfortable with the idea that determinations of ancillary care responsibilities were to be made by researchers on a case-by-case basis, and therefore an ‘ancillary care committee’ (a sub-committee of a research ethics committee) was built into the model. The model also – like the Belsky and Richardson model -- incorporates urgent care and the ‘duty to rescue’: when a research participant urgently needs medical help, it does not matter whether the condition is in the scope of the research; the researchers should respond as any human being would to the suffering and helplessness of another person.

A more detailed exposition of an alternative model of ancillary care responsibilities by workshop participants will be the subject of a future publication.

### Workshop message

The last day of the workshop was largely devoted to the crafting of a ‘workshop message’ addressed to interested parties in the DR Congo and abroad on the subject of ancillary care responsibilities in research. Participants were divided into three groups to discuss components of the message, and then representatives of the groups convened to compose a final statement (see Additional file 1). The final statement is to be presented to the National Health Ethics Committee of the DR Congo and to the DR Congo Ministry of Health. A distinguishing feature of the message is how specific duties related to ancillary care are placed on key stakeholders in the research enterprise, including prospective participants and their communities. In constructing the message, participants were surprised by the prevalence of ancillary care questions they experienced in local health research on the one hand, and the relative paucity of internationally recognized ethics guidance on the other.

## Discussion

The workshop brought together influential members of health-related agencies in the DR Congo to discuss an issue of common concern from an ethical perspective. Although the phenomenon of ancillary care needs in research was all too familiar to participants, they were less acquainted with the more theoretical aspects of the problem, as well as the complex ways that ancillary care is related to other issues such as the politics of research funding, global (health) inequalities, health and human rights, the duties of local health authorities, the responsiveness of research to local needs, how provision of ancillary care may negatively affect research, undue inducements in research, and so on. As the workshop progressed, it became obvious that tackling the problem of ancillary care involved engagement with a virtual cascade of interrelated ethical problems – both conceptual and practical -- at individual, community and global levels.

For this reason, we believe that the issue of ancillary care is an effective and appropriate entry point for engaging with research ethics in developing countries. Past capacity-building efforts in research ethics in developing countries have been criticized for overemphasizing certain topics or imposing foreign value frameworks [12,13]. Critics point out that the process of informed consent has been commonly been treated as the point of departure and central core of research ethics, both at home and abroad, to the neglect of other important ethical concerns. While consent is undoubtedly important, taking a consent-centered approach when teaching research ethics in developing countries has potential pitfalls: it may crowd out important local concerns, place excessive and culturally discordant value on individual decision-making, and lose sight of larger political, social and economic realities shaping health research. By focusing on ancillary care, and by building strongly interactive and hands-on components into workshops, some of these concerns can be mitigated. With ancillary care, there is little risk of discussing ethical questions that are not locally relevant. The issue permits participants to engage with conceptual questions – such as those of scope -- that have immediate practical implications. The topic also lends itself to the expression of personal experiences, worries and regrets that health advocates, policy-makers, field researchers or co-investigators may harbor, but have had no safe, ‘non-scientific’ forum in which to articulate them. Working collectively towards concrete outcomes, such as a workshop message, helps to facilitate a deeper appreciation of the complexities of research ethics.

Health-related research is becoming increasingly globalized, and it is important that competence in research ethics around the world keeps pace with this expansion. Resource-poor countries in particular need to be in a position to constructively criticize research that takes place in their communities from an ethical perspective. In settings where local capacity in research ethics is relatively undeveloped, it is crucially important to identify, share and implement appropriate and effective capacity-building models. We believe that an ancillary care-centered approach to research ethics capacity building is a promising contribution to this domain that could be usefully replicated in similar settings.

## Competing interests

The authors declare that they have no competing interests.

## Authors’ contributions

TT organized and informed the content of the original workshop on ancillary care and provided substantial input to the original (French) draft of the manuscript. SR wrote a preliminary outline of the manuscript, which was translated into French. BM was co-organizer of the workshop and facilitated the majority of the discussions.TT, BM, AM and PD provided substantial comments and additions to the French version of the manuscript, which was subsequently translated back into English and edited by SR. All authors agreed on the final content of the English version.

## Supplementary Material

Additional file 1 Statement issued by the workshop on the ethics of ancillary care in the context of research conducted in the Democratic Republic of Congo. Click here for file
