# Supplementary material for: Engaging with research ethics in central Francophone Africa: reflections on a workshop about ancillary care
Source: Philos Ethics Humanit Med. 2012 Aug 6;7:10. doi: 10.1186/1747-5341-7-10 (PMC3447659; doi:10.1186/1747-5341-7-10)
Supplement: Additional file 1 — Statement issued by the workshop on the ethics of ancillary care in the context of research conducted in the Democratic Republic of Congo. [file 1747-5341-7-10-S1.doc]

**STATEMENT ISSUED BY THE WORKSHOP ON THE ETHICS OF ANCILLARY CARE IN THE CONTEXT OF RESEARCH CONDUCTED IN THE DEMOCRATIC REPUBLIC OF THE CONGO**

We, the participants in the workshop on the ethics of ancillary care in the context of research performed in the Democratic Republic of the Congo, held at the Kinshasa/Gombe Rehabilitation Center for the Physically Disabled, on May 19-21 2011;

Whereas

- Ancillary care is a recent concept that is not yet well known and is not taken into account in research and intervention projects
- Funding for ancillary care in research projects is almost non-existent
- The vulnerability of our populations is a burden that hinders research in resource-poor countries
- National policy in regard to ancillary care (standards and guidelines) is currently non-existent
- Cultural perceptions of research projects can lead communities to believe that research efforts represent an opportunity to have all of their health problems addressed
- There is a high risk that research and intervention projects conducted in resource-poor countries will regularly encounter cases requiring ancillary care

Being aware that

- Ancillary care is a major problem in developing countries
- Few recognized international texts in the field of bioethics address this issue specifically or contextually

**WE RECOMMEND THAT:**

***The Government***

- Strengthen the healthcare system
- Develop standards and guidelines for ancillary care
- Launch research projects to assess the extent of ancillary care needs
- Mobilize financial resources to these ends

***Ethics Committees***

- Take into account the various aspects of ancillary care when reviewing research protocols
- Take into account cultural and socio-economic realities when defining conceptual and operational frameworks
- Disseminate the theoretical and practical frameworks for ancillary care
- Monitor the implementation and researchers' due consideration of ancillary care
- Consider remedial measures, where appropriate

***Researchers***

- Take into account the various aspects of ancillary care when designing, analyzing and implementing any research protocol,
- Budget funds for ancillary care in their research projects

***The wider community***

- Ensure that any proposed research addresses the issue of ancillary care,
- Effectively convey the need for ancillary care.

***Study participants***

- Understand that their participating in a study in no way obligates researchers to solve all of the problems in their lives

***The International Community***

- Incorporate ancillary care considerations into international declarations and agreements related to human subjects research
